# Cost-effectiveness of primary HPV genotyping and dual-stain or cytology reflex testing versus cytology-based screening for cervical cancer in Chile

**DOI:** 10.1371/journal.pone.0332010

**Published:** 2026-03-04

**Authors:** Nicolas Armijo, Manuel A. Espinoza, Pilar Contreras-Montiel, Macarena Vera, Carlos Balmaceda

**Affiliations:** 1 Epsilon Research, Santiago, Chile; 2 School of Public Health, Li Ka Shing Faculty of Medicine, The University of Hong Kong, China; National Cancer Institute, UNITED STATES OF AMERICA

## Abstract

Testing for high-risk human papillomavirus with genotyping for types 16 and 18 (HPV16/18) and triage by p16/Ki-67 dual-stain immunocytochemistry improves diagnostic performance in cervical cancer screening. We estimated the cost-effectiveness of HPV DNA–based primary screening strategies that detect high-risk genotypes every 5 years with either reflex cytology (hrHPV–Pap-5) or reflex dual stain (CINtec® PLUS, Roche; hereafter hrHPV–CINtec-5) versus cytology every 3 years (SoC (PAP-3)) among women aged 25–64 years, from the Chilean public healthcare perspective. A state-transition microsimulation reflected the natural history of cervical cancer in screening-eligible Chilean women, using local epidemiology and literature-informed inputs. Direct medical costs were obtained from official Chilean sources and converted to USD (1 USD = 938 CLP). Deterministic and probabilistic sensitivity analyses were conducted; a 30–64 years initiation scenario was also evaluated. Both high-risk HPV DNA-based strategies were more effective and cost-saving than SoC (PAP-3). In the 25–64 base case, hrHPV–CINtec-5 yielded the greatest health gain (13,003 incremental Quality-Adjusted Life Year, hereafter QALYs) with $16.65 saved per woman, while hrHPV–Pap-5 saved $32.57 with 12,844 QALYs. Probabilistic sensitivity analysis confirmed dominance (most simulations in the southeast quadrant) and cost-effectiveness acceptability >90% across willingness-to-pay ranges. Deterministic analysis highlighted progression risk from HPV16/18 and the discount rate as key drivers. Transitioning from PAP-3 to high-risk HPV DNA-based primary screening in Chile is projected to improve health outcomes while reducing costs. Among HPV DNA-based strategies detecting high-risk genotypes evaluated, triage with hrHPV–CINtec-5 provided the largest health gain while remaining cost-saving; hrHPV–Pap-5 maximized cost savings. These findings support modernizing the national screening program.

## Introduction

Cervical cancer is the fourth leading cause of death and incidence in women worldwide [1]. Persistent infection with human papillomavirus (HPV) has been identified as the major causal factor [2,3]. Although many HPV genotypes have been identified, HPV types 16 and 18 together cause about 70–73% of cervical cancers worldwide. [4]. Despite of significant progress in expanding access to HPV vaccination in many jurisdictions, cervical cancer incidence remains high [5]. Although HPV vaccination coverage has expanded, its full population-level impact is only now becoming evident as vaccinated cohorts age into higher-risk years. Screening therefore remains essential, and recommendations may evolve as high-coverage cohorts mature and use of the nonavalent vaccine expands [6,7].

In Chile, cervical cancer remains the seventh leading cause of death among women, despite a national screening programme in place for more than 28 years [8]. The programme—based on triennial cytology for women aged 25–64 years—, has never achieved the goal of 80% coverage target and declined markedly during the COVID-19 pandemic [9]. As an alternative to cytology as the primary screening method, the World Health Organization (WHO) recommends high-performance HPV DNA assays that detect a broad spectrum of carcinogenic high-risk types, while ensuring genotyping for HPV 16 and 18 (HPV16/18) which are implicated in over 70% of cervical cancers [10]. Compared with cytology, HPV DNA testing demonstrates higher sensitivity and specificity, be more reliable and reproducible because it does not depend on highly trained personnel and may increase coverage because of longer screening intervals (five years). Because many high-risk HPV infections clear spontaneously, triage of high-risk HPV-positive women is necessary; current strategies include HPV16/18 genotyping and repeat cytology [11]. Furthermore, the use of dual immunocytochemistry detecting co-expression of p16 and Ki-67 proteins (dual stain, DS), performed on liquid-based cytology slides prepared from the same cervical specimen collected for HPV testing [7] has demonstrated improved diagnostic accuracy among high-risk HPV-positive women, enhancing the triage process and the overall performance of the screening programme [11,12].

Compared to conventional cytology as a triage method, dual staining is associated with a lower risk of precancer within 3–5 years in negative cases and a higher risk in positive cases [7]. Evidence from the ATHENA study [13] shows that dual staining has greater sensitivity than cytology for detecting high-grade precancerous lesions, reducing unnecessary colposcopy referrals. Among HPV-positive women, it decreases colposcopy rates compared to cytology while increasing cervical intraepithelial neoplasia (CIN) grade 3 (CIN3+) detection, particularly in non-16/18 HPV cases, and also improves detection in 16/18-positive cases [14].

Current guidelines recommend dual staining in women positive for high-risk HPV non-16/18, referring positives to colposcopy and retesting negatives after one year [7,15]**.** After a negative dual-stain result, repeat HPV testing at 24 months is suggested; women who test HPV-negative return to routine screening, while those with persistent HPV positivity are managed according to the program’s triage/colposcopy pathway [16].

Although these technologies have higher upfront costs than cytology, determining significant additional resources in the short run. However, it is expected that a better diagnostic performance can generate significant savings over time, which motivated the study we report in this manuscript. This study aimed to estimate the cost-effectiveness of cervical cancer primary screening using high-risk HPV DNA testing every 5 years, with either reflex cytology (hrHPV–Pap-5) or dual-stain immunocytochemistry (hrHPV-CINtec-5), compared with cytology every 3 years (PAP-3), among women aged 25–65 years, from the perspective of the Chilean public healthcare system.

## Methods

### Natural history of disease model

**Fig 1:**
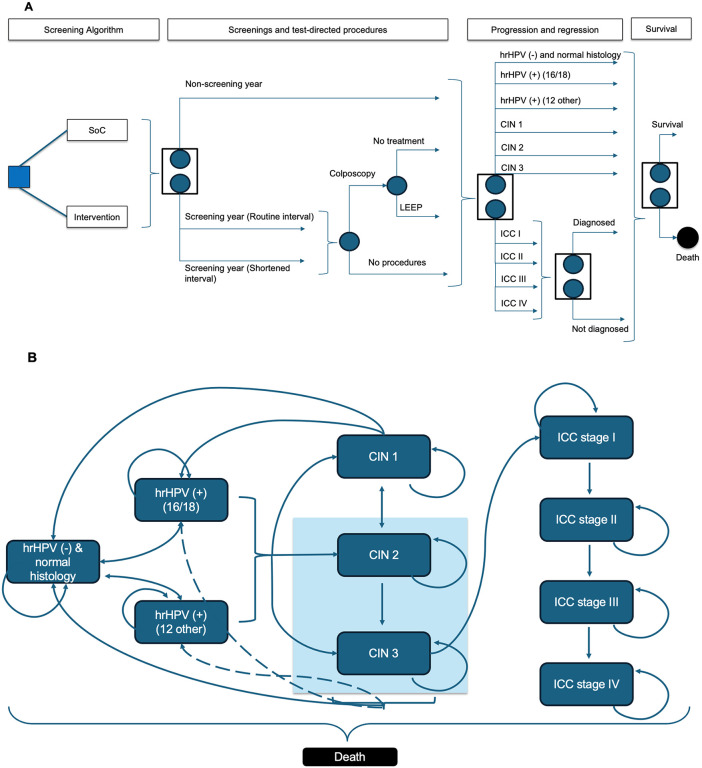
Decision Tree Diagram, Health States, and Annual Transitions Diagram. **A)** Decision tree diagram. The square represents a decision node, where a choice between strategies is made. The circle represents a chance node, capturing probabilistic events such as test outcomes or treatment success. The stacked symbols with arrows denote a Markov node, indicating transitions between health states in a Markov model; **B)** Health states and annual transition diagram. Solid arrows represent annual transitions between health states. Dashed arrows indicate transitions that occur only after a successful LEEP procedure, which is assumed to be applied in CIN 2 and CIN 3.The model simulates patients through multiple health states, representing high-risk HPV-negative with normal histology (no lesion); HPV-positive 16 or 18 (16/18) or 12 other high-risk genotypes (12 other+) – with normal histology; precancerous condition or HPV-positive with (CIN grade 1, 2, or 3, and invasive cervical cancer (ICC) – in any of its corresponding clinical stages, from stage I to **IV.** Patients were simulated under both the SoC (PAP-3) and the intervention strategy, enabling a within-subject comparison of clinical and economic outcomes.

A decision-analytic Markov microsimulation model with annual cycles and a lifetime horizon [17] was adapted from the perspective of the Chilean public healthcare system. The decision model was designed to reflect the natural history of cervical cancer disease and its long-term consequences, from model entry until death and to assess the impact of advanced HPV screening technologies (Fig 1).

### Comparators

The SoC in Chile was cervical cytology every three years without reflex testing, (PAP-3) [18]. This strategy was compared with primary high-risk HPV DNA testing using the cobas® 5800 system every five years with reflex dual-stained immunocytochemistry (p16/Ki-67; CINtec® PLUS) (hrHPV – CINtec-3). Additionally, PAP-3 was compared with a third strategy corresponding to high-risk HPV DNA testing every five years withreflex conventional cytology (hrHPV – PAP-5).

**Fig 2:**
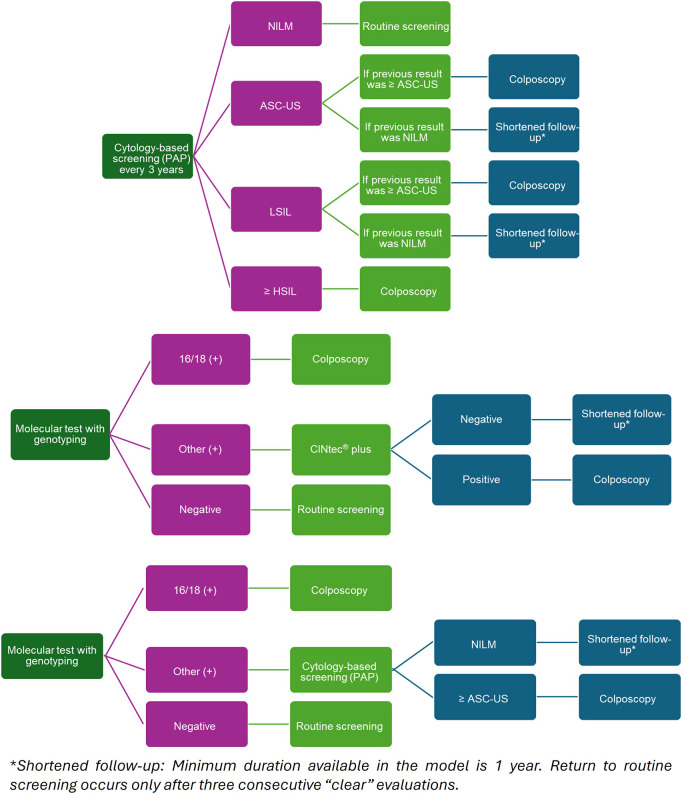
Screening algorithms for the PAP-3, hrHPV-CINtec, and hrHPV-PAP comparators. In the hrHPV–PAP-5 strategy, colposcopy is recommended for patients testing positive for HPV 16/18, or for 12 other+ when the reflex cytology result is ASC-US or higher. If the reflex cytology is NILM, shortened surveillance is advised. A negative test for high-risk HPV returns women to routine screening, with the next HPV DNA test scheduled in 5 years.

In the SoC algorithm, referral to colposcopy is indicated if the cytology result shows High-Grade Squamous Intraepithelial Lesion (HSIL) or a more severe lesion, or if the result is Low-Grade Squamous Intraepithelial Lesion (LSIL) or Atypical Squamous Cells of Undetermined Significance (ASC-US) with a prior cytology showing ASC-US (Fig 2). Shortened surveillance is recommended in cases where ASC-US or LSIL is identified following a previous Negative for Intraepithelial Lesion or Malignancy (NILM) result. If the result is NILM, the patient returns to routine screening, with cytology repeated in 3 years.

For the hrHPV–CINtec-5 strategy, patients with HPV 16/18 are referred directly to colposcopy. In cases of other high-risk HPV genotypes, CINtec® PLUS is used as a reflex test. If the CINtec® PLUS result is positive, colposcopy is recommended; if negative, shortened surveillance is indicated. As with the other strategies, a negative test for high-risk HPV results in return to routine screening with re-testing in 5 years.

### Model inputs

A detailed summary of all input parameters used in the cost-effectiveness analysis.

Is presented in Table 1. The following subsections describe the assumptions and data sources used for each model component.

**Table 1:** Model Input Base Case Values.

Input	Value	Range	Reference
hrHPV Test Sensitivity^a^	95.50%	91.10% − 99.00%	[17,19–21]
Cervical Cytology Test, % Results^b^			
Normal Histology			
NILM	71.10%	NA	[14,17,19]
ASCUS+	28.90%	8.10% − 36.10%	[14,17,19]
ASCUS	17.33%	NA	[14,17,19]
LSIL	9.76%	NA	[14,17,19]
HSIL	1.80%	NA	[14,17,19]
CIN-1			
NILM	39.96%	NA	[14,17,19]
ASCUS+	60.04%	45.00% − 75.10%	[14,17,19]
ASCUS	26.00%	NA	[14,17,19]
LSIL	31.50%	NA	[14,17,19]
HSIL	2.54%	NA	[14,17,19]
CIN-2			
NILM	40.11%	NA	[14,17,19]
ASCUS+	59.89%	44.90% − 74.90%	[14,17,19]
ASCUS	20.34%	NA	[14,17,19]
LSIL	27.97%	NA	[14,17,19]
HSIL	11.58%	NA	[14,17,19]
CIN-3			
NILM	27.23%	NA	[14,17,19]
ASCUS+	72.77%	54.60% − 91.00%	[14,17,19]
ASCUS	14.04%	NA	[14,17,19]
LSIL	13.62%	NA	[14,17,19]
HSIL	45.11%	NA	[14,17,19]
ICC			
NILM	14.29%	NA	[14,17,19]
ASCUS+	85.71%	64.30% − 100.00%	[14,17,19]
ASCUS	4.18%	NA	[14,17,19]
LSIL	6.27%	NA	[14,17,19]
HSIL	75.26%	NA	[14,17,19]
Dual Stain Results, % Positive			
No Lesions	30.90%	23.10% − 38.60%	[14,17,19]^c^
CIN-1	60.70%	45.50% − 75.80%	[14,17,19] ^c^
CIN-2	82.20%	61.70% − 100%	[14,17,19] ^c^
CIN-3	89.80%	67.30% − 100%	[14,17,19] ^c^
ICC	100%	75.00% − 100%	[14,17,19] ^c^
Colposcopy and Biopsy Sensitivity			
CIN-2	95.80%	91.40% − 99.00%	[22]
CIN-3	96.10%	91.40% − 100.0%	[22]
ICC, stage 1	100%	91.40% − 100.0%	[22]^f^
LEEP, % Success	100%	95.50% − 100.0%	[23]
Annual Health State Transition Probabilities			
From hrHPV-Negative & Normal Histology to			
hrHPV 16/18	1.00%	0.50% − 1.80%	[24]
hrHPV 12 Other	2.60%	2.00% − 3.20%	[24]
Fom hrHPV 16/18 to			
hrHPV-Negative & Normal Histology	29.70%	22.20% − 39.80%	[25]
CIN-1	6.20%	3.60% − 9.20%	[26]
CIN-2	2.20%	0.90% − 4.10%	[26]
CIN-3	2.10%	0.70% − 3.60%	[27]
From hrHPV 12 Other to			
hrHPV-Negative & Normal Histology	48.40%	37.30–58.20%	[25]
CIN-1	3.90%	1.60% − 6.90%	[26]
CIN-2	1.40%	0.10% − 3.40%	[26]
CIN-3	0.20%	0.10% − 0.30%	[27]
From CIN-1 to			
hrHPV-Negative & Normal Histology	27.60%	25.1% − 31.00%	[28,29]
hrHPV-Positive	3.10%	2.80% − 3.40%	[28,29]
CIN-2	8.20%	3.30% − 14.80%	[28]
CIN-3	1.80%	0.70% − 3.30%	[28]
From CIN-2 to			
hrHPV-Negative & Normal Histology	14.80%	5.10% − 28.40%	[28,30]
CIN-1	12.40%	4.30% − 23.70%	[28,30]
CIN-3	19.40%	2.50% − 49.00%	[28]
From CIN-3 to			
hrHPV-Negative & Normal Histology	10.20%	7.60% − 12.70%	[31]
CIN-1	4.10%	3.10% − 5.10%	[31]
ICC Stage 1	2.70%	1.70% − 4.50%	[31]
From ICC Stage 1–2	27.80%	20.80% − 34.70%	[32]
From ICC Stage 2–3	29.40%	22.10% − 36.80%	[32]
From ICC Stage 3–4	40.00%	30.00% − 50.00%	[32]
Cancer-Specific Mortality, Diagnosed ICC			
Stage 1	1.40%	1.20% − 1.50%	[33]
Stage 2	5.80%	5.20–6.40%	[33]
Stage 3	11.10%	10.40% − 11.80%	[33]
Stage 4	25.90%	24.40% 27.40%	[33]
Added Risk with Undiagnosed ICC	3.00%	2.30% − 3.80%	[34]
Probability of Symptoms with ICC			
Stage 1	7.50%	5.60% − 9.40%	[32]
Stage 2	11.30%	8.40% − 14.10%	[32]
Stage 3	30.00%	22.50% − 37.50%	[32]
Stage 4	45.00%	33.80% − 56.30%	[32]
Quality of Life			
Utility without ICC Diagnosis	0.90	0.84–0.95	[35]
Disutility with ICC Diagnosis Initial Phase, by Stage of Diagnosis^c^			
Stage 1	0.07	0.02–0.12	[35]^e^
Stage 2	0.19	0.03–0.36	[35]^e^
Stage 3	0.19	0.03–0.36	[35]^e^
Stage 4	0.19	0.03–0.36	[35]^e^
Continuing Phase^d^	0.08	0.05–0.11	[35]
Terminal Phase, Cancer Death^d^	0.54	0.50–0.57	[35]
Annual Costs			
Cancer Costs Initial Phase			
Stage 1	3,593	2,694–4,491	[36–38] ^c^
Stage 2	4,158	3,119–5,198	[36–38] ^c^
Stage 3	6,013	4,509–7,516	[36–38] ^c^
Stage 4	5,661	4,246–7,077	[36–38] ^c^
Cancer Costs Continuing Phase			
Stage 1	97	73 - 122	[36,37] ^c^
Stage 2	97	73 - 122	[36,37] ^c^
Stage 3	241	180 - 301	[36,37] ^c^
Stage 4	241	180 - 301	[36,37] ^c^
Cancer Costs Terminal Phase			
Stage 1	1,634	1,226–2,043	[36–39] ^c^
Stage 2	1,634	1,226–2,043	[36–39] ^c^
Stage 3	1,634	1,226–2,043	[36–39] ^c^
Stage 4	1,634	1,226–2,043	[36–39] ^c^
Non-Cancer Death			
Stage 1	1,289	967–1,611	[36–39] ^c^
Stage 2	1,289	967–1,611	[36–39] ^c^
Stage 3	1,289	967–1,611	[36–39] ^c^
Stage 4	1,289	967–1,611	[36–39] ^c^
Procedure Costs			
Primary Care Visit	10.33	8 - 13	[37] ^c^
hr-HPV Test with Genotyping	13.86	10 - 17	^c f^
Cervical Cytology Test	7.14	5 - 9	[37] ^c^
Dual Stain Cytology	37.31	28 - 47	^c, g^
Colposcopy and Biopsies	46.76	35 - 58	[37] ^c^
Histopathological Analysis of Cervical Biopsy Post-Colposcopy	14.57	11 - 18	[37] ^c^
LEEP	306.60	230 - 383	[36,37] ^c^
Histopathological Analysis of Cervical Biopsy Post-LEEP	28.92	22 - 36	[37] ^c^
Annual Discount Rate			
Costs	3%	0% − 5%	[40]
QALYs	3%	0% − 5%	[40]

hrHPV: High-Risk Human Papillomavirus types. CIN-1: Cervical Intraepithelial Neoplasia grade I. CIN-2: Cervical Intraepithelial Neoplasia grade II. CIN-3: Cervical Intraepithelial Neoplasia grade III. ICC: Invasive Cervical Cancer. NILM: Negative for Intraepithelial Lesion or Malignancy. ASCUS + : Atypical Squamous Cells of Undetermined Significance or higher. ASCUS: Atypical Squamous Cells of Undetermined Significance. LSIL: Low-Grade Squamous Intraepithelial Lesion. HSIL: High-Grade Squamous Intraepithelial Lesion. NA: Not applied. LEEP: Loop Electrosurgical Excision Procedure. QALY: Quality-Adjusted Life Year. ^a^: For the presence of hrHPV. ^b^: Cervical cytology (distribution of results by histology). Only the composite ASCUS+ proportion carries uncertainty; the shares for NILM, ASCUS, LSIL, and HSIL are fixed to trial distributions and were not varied independently. In sensitivity analyses, ASCUS+ was varied over its reported range and NILM was adjusted so that category probabilities sum to 100% within each histology stratum; the internal split of ASCUS, LSIL, and HSIL remained fixed. ^c^: Range established as +/- 25% of the base case value. ^d^: The terminal phase refers to the year before death, the initial phase corresponds to the year following diagnosis (excluding the terminal phase), and the continuing phase covers any intervening time between the initial and terminal phases. ^e^: 95% confidence interval, calculated as the annual average (for stage 1–4 cervical cancer in the initial phase). ^f^: Includes three biopsies and pathology at base case, range 1–4 biopsies, per the Biopsy Study [22]^g^: Provided by the company.

#### Target population.

We modelled a synthetic cohort of women aged 25 years and older who use Chile’s public health system and are eligible for routine cervical cancer screening. In line with the national program, routine screening is targeted to women aged 25–64 years in the general population [41,42]. Women with history of CIN 1, 2 or 3, ICC, Human Immunodeficiency Virus diagnosis, total hysterectomy, or pregnancy were excluded.

At baseline, the synthetic cohort was initialized as a set of mutually exclusive age–genotype–histology strata: age groups (25–29, 30–39, 40–49, 50–59, 60–65), hr-HPV genotypes (16/18**,** 12 other + **,** negative), and histologic states (no lesion, CIN1, CIN2, CIN3, ICC). Each stratum received a weight equal to its proportion among screening-eligible women. Model outcomes were computed as the weighted sum across strata. In each annual cycle, the model generated new screening-eligible entrants using the same age structure to maintain a stable age distribution over time.

Chile’s cervical screening delivery is predominantly opportunistic (i.e., no national call–recall system), and historical coverage has remained below the 80% program target, which contextualizes our assumption of equal adherence across strategies [9].

Prevalence data for high-risk HPV genotypes, HPV 16/18 and 12 other + , were obtained from literature with local data [43] and disaggregated by age groups to match model inputs (Table 2). Histological lesion prevalence, including no lesions, CIN 1–3 and ICC, was stratified by age and genotype, based on national sources and REM (Registro Estadístico Mensual) data [44]. Additional details on the prevalence estimations are available in the supporting information (S1 and S2 Files).

**Table 2:** Model Initialization Based on High-Risk HPV types and Histopathological Status.

Age group	Genotype	hrHPV Prevalence by age	Histological Status prevalence by Age and hrHPV genotype					
			Normal	CIN −1	CIN-2	CIN-3	ICC	Total
25–29	16/18	6.1%	74.82%	18.21%	4.67%	2.18%	0.11%	100%
	12 Other	19.5%	89.79%	7.51%	1.82%	0.85%	0.02%	100%
30 - 39	16/18	3.3%	76.55%	14.67%	4.78%	2.97%	1.04%	100%
	12 Other	10.5%	90.86%	5.98%	1.84%	1.15%	0.17%	100%
40–49	16/18	2.6%	81.87%	11.15%	3.45%	2.12%	1.41%	100%
	12 Other	8.2%	93.34%	4.37%	1.28%	0.78%	0.23%	100%
50–59	16/18	3.1%	87.42%	7.89%	1.87%	1.29%	1.53%	100%
	12 Other	9.8%	95.67%	2.97%	0.66%	0.46%	0.23%	100%
60 - 65	16/18	3.3%	88.97%	7.89%	1.87%	1.29%	1.53%	100%
	12 Other	10.3%	96.31%	2.43%	0.59%	0.43%	0.24%	100%

hrHPV: high-risk human papillomavirus types. CIN-1: Cervical Intraepithelial Neoplasia grade I. CIN-2: Cervical Intraepithelial Neoplasia grade II. CIN-3: Cervical Intraepithelial Neoplasia grade III. ICC: Invasive Cervical Cancer. Estimates are presented in the Supporting Information. Sensitivity analyses explored ±25% variations in the overall prevalences of hrHPV 16/18, other 12 hrHPV types, and associated lesions by genotype.

#### Transition probabilities.

Transition probabilities between health states in the Markov model were obtained from a range of sources, including clinical trials and observational studies considered as real-world evidence, all derived from international publications [24–32].

Disease progression across ICC stages (from stage I to stage IV) in undiagnosed patients, as well as the probability of symptoms onset among individuals with ICC, was informed by previously published data [32,34]. It was assumed that once symptoms appeared, patients were diagnosed with ICC. Cancer-specific mortality by ICC stage was estimated using survival data based on US cancer-specific survival data from the Surveillance, Epidemiology, and End Results cancer survival data [33].

#### Utilities.

Health-related quality of life (HRQoL) was incorporated into the model through utility values assigned to health states defined in the decision model, where a value of 1 represents perfect health and 0 represents death. A baseline utility of 0.90 was applied to individuals without a diagnosis of ICC, collapsing all cytology results into a single health state for the screening population (Table 1). For individuals with ICC, stage- and phase-specific disutilities were applied based on de Kok et al**.**: for the initial year, EuroQol 5 Dimensions (EQ-5D) values at diagnosis, 3, 6, and 12 months were combined into a time-weighted annual mean (1.5/3/4.5/3 months), and the disutility was the difference from 0.90 (0.07 for International Federation of Gynecology and Obstetrics (FIGO) stage I; 0.19 for FIGO stages II–IV); during the continuation phase (tumour-free years 2–10), EQ-5D 0.82 implied a disutility of 0.08 (95% CI 0.05–0.11); for the terminal year, EQ-5D 0.36 implied a disutility of 0.54 (95% CI 0.50–0.57) [35].

#### Healthcare resource utilisation and costs.

Costs included direct medical costs related to screening tests, diagnostic and clinical procedures, and cervical cancer management across its different stages. To estimate these costs, resource use baskets were developed to reflect the services typically provided in the Chilean public system from initial screening through to cancer treatment. Unit cost sources included the 2021 National Cost Verification Study (Estudio de Verificación de Costos, EVC) [36], the Central Supply Center of the National Health Services System (CENABAST) [38], the 2024 Chile’s National Health Fund (FONASA) Institutional Tariff [37], and Chile’s public procurement platform (Mercado Público) [39]. Costs related to screening comparators (cobas® and CINtec® PLUS) were provided by the company.

All costs were estimated in Chilean pesos (CLP) from the perspective of the public healthcare system and converted into United States Dollars (USD) using an exchange rate of 1 USD = 938 CLP. An undifferentiated annual discount rate of 3% was applied to both costs and health at the base case and analysis scenario.

#### Outcomes.

The performance of cervical cancer screening tests was informed by the results of the IMPACT trial and supporting published literature [14,17,19,22]. Cervical cytology performance was estimated by the health status of patient (normal histology, CIN 1–3), and the results of sensitivity were expressed for the five possible results: NILM, ASC-US, LSIL or HSIL.

The sensitivity of high-risk HPV detection in this analysis (Cobas® 5800 system) was derived from the IMPACT Trial; although it delivers the performance of Cobas® 4800, it has been validated in a previous study that both have comparable clinical validity [20,21]. Performance of CINtec ® Plus was informed by the IMPACT trial as well and was expressed in terms of the probability of a positive CINTec® Plus result with normal histology or with abnormal histology for CIN1–3 or ICC.

Colposcopy and biopsy sensitivity was derived from Wentzensen et al [22], which reported performances over 90% and assumed 100% sensitivity for detecting ICC in stage 1.

The quality-adjusted life-years (QALY), and total costs for SoC and intervention arm were calculated in each cycle of the simulation. Results were expressed as incremental cost, incremental QALY, cost per QALY gained (incremental cost-effectiveness ratios, ICER), and population-level incremental QALY. Per capita gross domestic product (GDP) of Chile in 2023 (US$17,093/QALY according to World Bank) was used as the willingness-to-pay (WTP) threshold to assess the cost-effectiveness of competing treatments.

### Sensitivity analyses

For the deterministic sensitivity analysis (DSA), each parameter was varied independently (ceteris paribus), either across its reported confidence interval or by ±25% from its base-case value. Results are presented using a tornado diagram to illustrate the univariate impact of each parameter on the model outcomes. The probabilistic sensitivity analysis (PSA) aimed to capture parameter uncertainty by jointly varying all parameters based on their assigned probability distributions. A total of 1,000 Monte Carlo simulations were performed. The results are presented as cost-effectiveness scatter plots and cost-effectiveness acceptability curves (CEACs).

It is worth noting that bootstrap 95% CIs were calculated to assess the impact of patient variability using 1,000 bootstrap samples of 100,000 microsimulated patients; these values were presented in the incremental results.

### Analysis scenario

The base case considered for HPV screening was a target population of women aged 25–65 years who met the inclusion criteria described previously. The cohort received either SoC, corresponding to PAP-3, or one of the alternative strategies that were compared with the SoC: hrHPV-PAP-5 or hrHPV-CINTec-5. Adherence of 100% to screening, treatment and follow-up was assumed.

Additionally, a scenario analysis was conducted in which the target population was modified to include women aged 30–65 years. All other parameters remained identical to those in the base case.

## Results

Compared to the SoC (PAP-3), both intervention strategies, hrHPV-PAP-5 and hrHPV-CINtec-5, were associated with a reduction in total costs and an improvement in health outcomes (Table 3). On a per-patient basis, the strategy combining high-risk HPV genotyping with cytology reflex testing (hrHPV-PAP-5) showed the greatest reduction in total costs, saving an average of $32.57 (95% CI: –$35 to –$30), while the hrHPV–CINtec-5 strategy saved $16.65 (95% CI: –$20 to –$13). These savings were primarily driven by reductions in ICC and fewer screening visits, despite slightly higher costs associated with screening tests, colposcopies, and treatment of precancerous lesions (CIN 1–3). In terms of health outcomes, hrHPV-CINtec-5 strategy achieved the highest population-level benefit, producing an estimated 13,003 incremental QALYs (95% CI: 6,403–20,632), slightly above the 12,844 QALYs gained with the hrHPV–PAP-5 strategy (95% CI: 6,272–20,643). At the individual level, the incremental QALYs were similar between strategies, at 0.0046 and 0.0045, respectively. Both interventions resulted in negative Incremental Cost-Effectiveness Ratios (ICERs) (–$7,191 and –$3,632 per QALY), confirming their dominance.

**Table 3:** Incremental Results.

Change per average patient with intervention instead of SoC	SoC vs hrHPV-CINtec-5		SoC vs hrHPV-PAP-5	
Base case				
	Mean	95% CI	Mean	95% CI
Costs				
Screening Visits	-$49.70	(-$50 - -$49)	-$49.87	(-$51 - -$49)
Screening Tests	$17.18	($16 – $18)	$2.22	($2 - $3)
Colposcopies^a^	$13.77	($13 – $15)	$13.15	($12 - $14)
Treatments^a^	$6.54	($6 – $7)	$5.92	($5 - $7)
Invasive Cervical Cancer	-$4.44	(-$7 - -$3)	-$3.99	(-$6 - -$2)
Total	-$16.65	(-$20 – $13)	-$32.57	(-$35 - -$30)
QALYs	0.0046	(0.0023–0.0073)	0.0045	(0.0022–0.0073)
Cost per QALY	-$3,632	(-$7,208 - -$2,340)	-$7,191	(-$15,342 - -$4,599)
Population-level incremental QALY^b^	13,003	(6,403–20,632)	12,844	(6,272–20,643)
Scenario analysis 1				
	Mean	95% CI	Mean	95% CI
Costs				
Screening Visits	-$47,67	(−48 - −47)	-$47,82	(−48 - −47)
Screening Tests	$12,13	(11 - 13)	-$0,73	(−1 - −0,057)
Colposcopies^a^	$10,47	(9 - 12)	$9,93	(9 - 11)
Treatments^a^	$5,25	(5 - 6)	$4,73	(4 - 5)
Invasive Cervical Cancer	-$4,96	(−8 - −3)	-$4,75	(−7 - −2)
Total	-$24,79	(−29 - −22)	-$38,62	(−42 - −35)
QALYs	0,0052	(0.0024–0.0090)	0.0051	(0.0023–0.0088)
Cost per QALY	-$4.731	(-$10,216 - -$2,701)	-$7,632	(-$17,110 - -$4,362)
Population-level incremental QALY^b^	12,852	(5,915–22,190)	12,415	(5,591–21,664)

SoC: cervical cytology every 3 years. hrHPV-CINtec-5: HPV genotyping and dual stain cytology every 5 years. hrHPV-PAP-5: HPV genotyping and cervical cytology every 5 years. ^a^: Iincludes biopsy and pathology costs. ^b^: Target population aged 25 and over: 2,835,996; aged 30 and over: 2,453,048.

When screening was initiated at age 30 instead of 25, both intervention strategies continued to dominate the SoC (PAP-3), generating cost savings and improved health outcomes. The hrHPV-PAP-5 showed the greatest total cost reduction, with savings of $38.62 per patient (95% CI: –$42 to –$35), compared to $24.79 (95% CI: –$29 to –$22) for the hrHPV–CINtec–5 strategy. Both interventions remained cost-saving, with negative ICERs of –$7,632 and –$4,731 per QALY, respectively. At the population level, the hrHPV-CINtec-5 strategy produced the highest health benefit, with 12,852 incremental QALYs (95% CI: 5,915–22,190), while the hrHPV–PAP-5 strategy yielded 12,415 QALYs (95% CI: 5,591–21,664).

**Fig 3:**
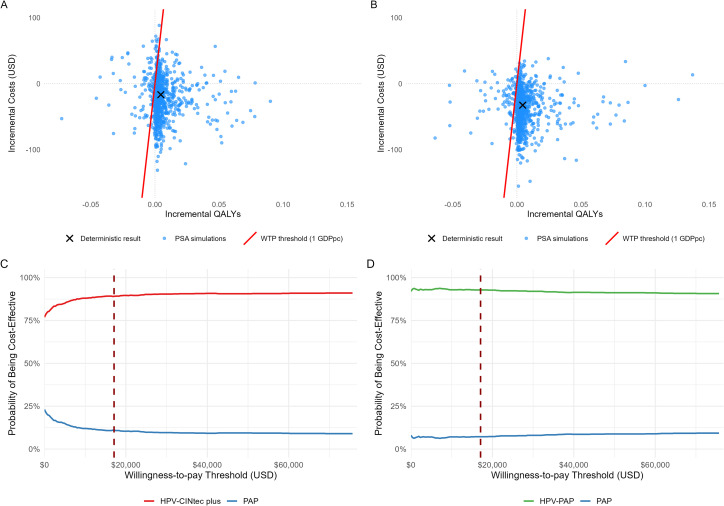
Probabilistic Sensitivity Analysis Results for Cervical Cancer Screening Strategies. **A)** Scatter plot of PSA simulations for the hrHPV-CINtec-5 strategy versus PAP-3; **B)** Scatter plot for the hrHPV-PAP-5 strategy versus PAP-3; **C)** Cost-effectiveness acceptability curve (CEAC) corresponding to panel **(A)**; **D)** CEAC corresponding to panel **(B)**. Dashed vertical lines represent the willingness-to-pay threshold set at one GDP per capita (USD$17.093 according to World Bank).

Fig 3 presents the outcomes of the PSA through cost-effectiveness panels (Panels A and B) and CEACs (Panels C and D) for the two alternative screening strategies. Panel A shows the incremental costs and QALYs of the hrHPV-CINtec-5 versus PAP-3. Most PSA simulations fall in the southeast quadrant, indicating that this strategy is both more effective and less costly than the SoC (PAP-3). Complementarily, the CEAC (Panel C) shows that the hrHPV-CINtec-5strategy maintains a high probability of being cost-effective across a wide range of willingness-to-pay (WTP) thresholds, surpassing 75% even at low thresholds and exceeding 90% as the threshold increases. Similarly, the HPV-PAP-5 strategy was more effective and consistently less costly than SoC (PAP-3) in most simulations (Panel B). The CEAC (Panel D) further supports these findings, showing a high probability of cost-effectiveness across the entire range of WTP thresholds.

**Fig 4:**
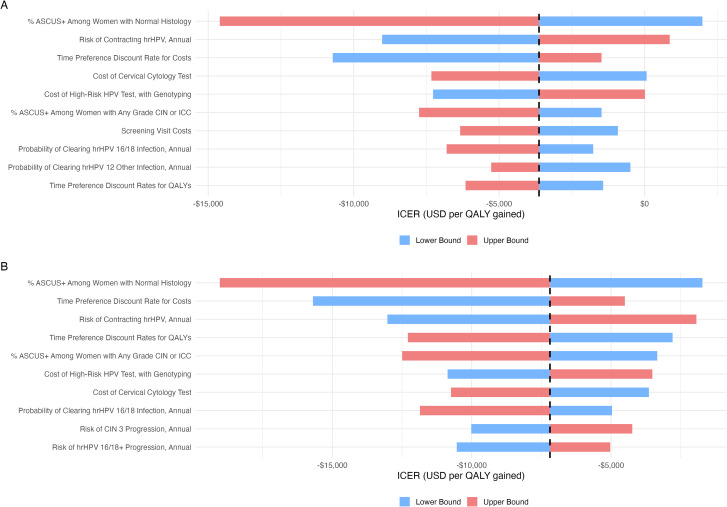
One-Way Sensitivity Analysis (OWSA). A) hrHPV-CINtec-5 strategy versus PAP-3; B) hrHPV-PAP-5 strategy versus PAP-3. Dashed vertical lines represent the willingness-to-pay threshold set at one GDP per capita (USD$17.093 according to World Bank).

Fig 4a presents the DSA comparing hrHPV-CINtec-5against conventional PAP-3. The most influential parameter was the false-positive rate of ASCUS+ among women with normal histology, with an ICER range from –$14,603 to $1,982 per QALY gained. Other key drivers of uncertainty included the annual risk of acquiring hr HPV (–$9,023 to $861) and the discount rate for costs (–$10,721 to –$1,484). Despite the wide variation introduced by these parameters, the ICER remained negative in most cases, consistently favouring the molecular-based strategy over PAP-3.

Fig 4b displays the DSA for the comparison between hrHPV-PAP-5 versus PAP-3. In this scenario, the ASCUS+ rate among women with normal histology again showed the largest impact (–$19,041 to –$1,722), followed by the discount rate for costs (–$15,698 to –$4,504) and the risk of acquiring hr HPV (–$13,025 to –$1,937). All univariate variations resulted in ICERs below zero, reinforcing the conclusion that molecular screening strategies are economically dominant compared to conventional cytology, even under conservative assumptions.

## Discussion

Our findings indicate that introducing primary HPV DNA testing with genotyping for types 16 and 18, either followed by reflex cytology (hrHPV–Pap-5) or dual-stain immunocytochemistry (e.g., CINtec® PLUS or other p16/Ki-67 assays; hrHPV–CINtec-5), is expected to significantly surpass the existing cytology-only screening method (PAP-3) in Chile.

Both molecular-based strategies not only improve health outcomes but also reduce overall costs compared to the status quo. In the base-case analysis, hrHPV-Pap-5 yielded an average savings of $32.6 per woman, while hrHPV-CINTec-5 resulted in $16.65 in savings. Both methods provided roughly 0.0045 QALYs per individual over a lifetime, leading to substantial benefits at the population level, approximately 13,000 QALYs gained for hrHPV–CINtec-5, alongside net cost reductions. The resulting ICERs were negative, confirming that both strategies are cost-saving.

These results are robust across a wide range of assumptions. Probabilistic sensitivity analysis showed that most simulation iterations for hrHPV–CINtec-5 fell in the southeast quadrant of the cost-effectiveness plane, indicating greater effectiveness at lower cost. Correspondingly, the cost-effectiveness acceptability curve for the dual-stain strategy remains high across all WTP levels, exceeding a 90% probability of being cost-effective at even modest thresholds. In other words, the likelihood of adopting a non-cost-effective strategy is exceptionally low.

Deterministic sensitivity analyses further supported this finding: variations in progression risks, test performance, or discount rate did not alter the conclusion of dominance. The consistency of results confirms that the superiority of high-risk HPV DNA-based screening is not driven by specific assumptions but is a stable outcome given the data and model structure.

For instance, even when the progression risks, test characteristics, or discount rate were stress-tested, the ICER for high-risk HPV DNA testing remained negative in all scenarios examined. This consistency suggests that our conclusion is not an artefact of assumptions, but a reliable outcome given the data and structure of the model.

The economic advantage for high-risk HPV DNA-based strategies is primarily driven by their superior clinical performance and overall efficiency. Using a more sensitive test upfront, more precancerous lesions are detected and treated before progressing to invasive cancer, which averts costly cancer treatments down the line. Indeed, our model attributes the cost savings primarily to a reduction in expensive invasive cancer cases and a decrease in the number of screening visits (due to longer screening intervals), more than compensating for the higher unit costs of high-risk HPV testing, immunocytochemical triage, additional colposcopies, and treatment of additional precancerous lesions.

The p16/Ki-67 dual-stain triage () contributes to cost containment by improving risk stratification. Evidence from a large U.S. screening programme showed that p16/Ki-67 dual-stain triage can safely replace cervical cytology, yielding equal or better detection of high-grade lesions while referring approximately 32% fewer women to colposcopy [12]. This improved specificity means fewer unnecessary procedures and follow-ups for women without significant disease, which not only reduces direct medical costs but also spares women the anxiety and morbidity associated with overtreatment.

Our model reflects these benefits: the hrHPV - CINtec-5 strategy achieved slightly greater health gains than hrHPV - Pap-5 (preventing more cervical cancer cases), while substantially streamlining the diagnostic work-up. The ability to maintain high sensitivity for detecting precancer, demonstrated in clinical trials (Wentzensen et al.’s findings) [12]. The ability to maintain high sensitivity for precancer (as demonstrated in clinical trials) and simultaneously avoid redundant colposcopies underpins the dual-stain strategy’s robust performance on both health and economic fronts.

It is informative to compare our results with those reported in other settings. Harper et al. recently evaluated adding p16/Ki-67 dual-stain cytology in a U.S. co-testing context (where HPV and cytology are both used as primary tests) and found it to be a cost-effective enhancement, with ICERs on the order of $10,000–$23,000 per QALY gained [17]. That study assumed a more resource-intensive baseline (co-testing with no biomarker triage), which yielded incremental improvements from dual-stain rather than outright cost savings. In contrast, our analysis examined a setting where the current standard is PAP-3 screening, which is less effective at early detection. This difference in baseline explains why incorporating HPV testing (with or without dual stain) in Chile appears even more attractive: there is a larger margin for improvement over cytology screening, leading to net savings as future cancer cases (and their treatment costs) are averted. The value of advanced HPV screening technologies may be even greater in health systems that have yet to transition away from cytology. Our findings align with other studies in middle-income countries showing that HPV-based screening can be “very cost-effective” or even cost-saving compared to cytology-based strategies [45], thanks to the higher sensitivity of HPV testing and the efficiency of longer screening intervals. Moreover, the dual-stain reflex test –approved and recommended in various jurisdictions as a triage for HPV-positive women [7,15,46–48] – has demonstrated superior accuracy in identifying clinically relevant lesions than conventional cytology triage [12]. By capitalising on these performance gains, the Chilean programme can expect not only improved cancer prevention outcomes but also a more efficient allocation of resources.

In Latin America, findings from the ESTAMPA consortium support the programmatic superiority of primary HPV screening over cytology and provide standardized evidence on triage and colposcopy performance; these regional data are directionally consistent with our results and reinforce the policy case for adopting HPV-based screening [49–51].

An important strength of this study is the use of Chilean real-world data and a consistent modelling framework. We populated the microsimulation with local epidemiological inputs, including age-stratified prevalence of HPV (by genotype) and cervical lesion probabilities derived from Chilean sources. Likewise, costs for screening tests, follow-up procedures, and cancer care were taken from the Chilean public payer perspective. Grounding the model in local data enhances the validity of the results for national decision-making, ensuring that the projected outcomes reflect the realities of Chile’s population and healthcare system. We also took care to align all model parameters to a common reference standard – effectively, all probabilities and accuracies are conditional on the same underlying disease definitions. This avoids the potential bias that can occur if, for example, disease prevalence is estimated using one definition (or gold-standard test) while test sensitivity/specificity is measured against a different or imperfect standard [52]. In diagnostic modelling, a mismatch between the target condition and the reference used to derive test performance can distort results [52]. By ensuring internal consistency (i.e., that the “case” definition for cervical neoplasia is uniform across prevalence estimates, test characteristics, and outcomes), our analysis conforms to best practices and recent recommendations in the field [52]. This methodological rigor adds confidence that the dominance of the HPV-based strategies is not an artefact of inconsistent assumptions.

Several practical considerations emerge from this analysis. First, although the model projects net cost savings over a lifetime horizon, implementing HPV-based screening will require upfront investment – for instance, in testing equipment, laboratory capacity, and provider training. Decision-makers should be prepared for higher screening expenditures in the short term, even though these are offset by downstream savings. Our results suggest that these investments are sound given that, over a lifetime horizon, both HPV strategies are cost-saving relative to PAP-3. Second, we assumed in our base case that women adhere to screening and follow-up recommendations. Chile’s historical cytology programme has faced suboptimal coverage (never reaching the target 80% of eligible women) and losses to follow-up. The superior test characteristics of high-risk HPV DNA testing and the possibility of self-collection could improve participation rates over time, but realising the full health gains we project will depend on strengthening outreach and ensuring that HPV-positive women complete their triage and treatment. Any gaps in the screening continuum could dilute the anticipated benefits. Although longer screening intervals (e.g., 5 vs 3 years) may influence attendance, the empirical evidence is mixed and likely context dependent [53,54]. We therefore treat equal adherence as a conservative assumption for HPV DNA-based primary screening strategies that detect high-risk genotypes: if longer intervals ultimately improve adherence, our incremental health gains and cost offsets are likely underestimated.

Third, a further methodological limitation concerns health-state valuation. We did not use Chilean or Latin-American utility weights; instead, HRQoL parameters were drawn from international sources. Further research is needed to estimate HRQoL at the national or regional level. Once region-specific utility estimates become available, the model can be updated by substituting those utilities and re-estimating outcomes. In the meantime, we propagated utility uncertainty in both DSA and PSA; the incremental results and the preferred strategy remained unchanged, indicating that our conclusions are robust to plausible uncertainty in utility values.

Lastly, while our model captures a comprehensive range of costs from the public healthcare perspective, it does not quantify broader societal benefits. Reducing cervical cancer cases has implications beyond direct medical costs – for example, averting productivity losses and the profound social impact on patients and families. Thus, the true value of preventing cervical cancer likely exceeds even what our conservative health system analysis demonstrates.

In summary, this cost-effectiveness study provides strong evidence in support of modernising the cervical cancer screening programme in Chile. Transitioning from cytology every three years to high-risk human papilloma virus (HPV) testing, whether through HPV with genotyping for types 16/18 with cytology reflex every five years or with p16/Ki-67 dual-stain immunocytochemistry (CINtec Plus^®^) every five years, for women aged 25–64, offers a win–win: better health outcomes for Chilean women at a lower overall cost to the health system.

Both evaluated HPV-based algorithms proved unequivocally cost-effective, and in fact cost-saving, compared to the current standard. Among the two, HPV testing with genotyping and reflex p16/Ki-67 dual-stain immunocytochemistry achieved the greatest health benefit, preventing the most cervical cancers, while still substantially reducing costs relative to the status quo. This finding aligns with the WHO recommendation to adopt high-performance HPV screening technologies to accelerate cervical cancer prevention [55,56]. By leveraging a more sensitive primary test and an objective, biomarker-based triage, Chile can expect to improve the early detection of precancerous lesions and reduce the burden of invasive disease. Importantly, our analysis suggests that these clinical improvements need not break the budget – on the contrary, they free up resources in the long term. Given the robustness of these results across uncertainty analyses, policymakers can be confident that investing in HPV screening with reflex immunocytochemistry is a sound and evidence-based strategy. Such a reform would not only contribute to national health objectives but also support the global effort to eliminate cervical cancer as a public health problem. The challenge now lies in implementation, but the economic and health rationale for change is compelling. The Chilean public healthcare system stands to gain a more effective, efficient, and impactful screening programme by embracing these advancements in cervical cancer prevention.

## Supporting information

S1 File Estimation of age-specific prevalence of HPV genotypes. S1 describes the estimation of age-specific high-risk HPV prevalence based on Chilean national data and its disaggregation into HPV 16/18 and other high-risk genotypes.
(DOCX)

S2 File Estimation of histological lesions by genotype and age. S2 describes the estimation of age- and genotype-specific distributions of cervical histological lesions by combining published genotype proportions with Chilean administrative data and mapping Bethesda classifications to CIN categories.
(DOCX)

## References

[1] Wu J, Jin Q, Zhang Y, Ji Y, Li J, Liu X. Global burden of cervical cancer: Current estimates, temporal trend and future projections based on the GLOBOCAN 2022. Journal of the National Cancer Center. 2025. 10.1016/j.jncc.2024.11.006 PMC12276544 40693230

[2] WHO. Cervical Cancer: WHO; 2025 https://www.who.int/health-topics/cervical-cancer#tab=tab_1

[3] Walboomers JM, Jacobs MV, Manos MM, Bosch FX, Kummer JA, Shah KV, et al. Human papillomavirus is a necessary cause of invasive cervical cancer worldwide. J Pathol. 1999;189(1):12–9. doi: 10.1002/(SICI)1096-9896(199909)189:1<12::AID-PATH431>3.0.CO;2-F 10451482

[4] de Martel C, Plummer M, Vignat J, Franceschi S. Worldwide burden of cancer attributable to HPV by site, country and HPV type. Int J Cancer. 2017;141(4):664–70. doi: 10.1002/ijc.30716 28369882 PMC5520228

[5] Rahangdale L, Mungo C, O’Connor S, Chibwesha CJ, Brewer NT. Human papillomavirus vaccination and cervical cancer risk. BMJ. 2022;379:e070115. doi: 10.1136/bmj-2022-070115 36521855

[6] Joura EA, Giuliano AR, Iversen O-E, Bouchard C, Mao C, Mehlsen J, et al. A 9-valent HPV vaccine against infection and intraepithelial neoplasia in women. N Engl J Med. 2015;372(8):711–23. doi: 10.1056/NEJMoa1405044 25693011

[7] World Health Organization (WHO). WHO Guideline for screening and treatment of cervical pre-cancer lesions for cervical cancer prevention: Use of dual-stain cytology to triage women after a positive test for human papillomavirus (HPV) [Internet]. 2024. Geneva. https://www.who.int/publications/i/item/9789240091658 38976622

[8] ICO/IARC Information Centre on HPV and Cancer (HPV Information Centre). Chile. Human Papillomavirus and Related Cancers, Fact Sheet 2023 2023 https://hpvcentre.net/statistics/reports/CHL_FS.pdf

[9] Bruni L, Albero G, Serrano B, Mena M, Collado J, Gómez D. Human Papillomavirus and Related Diseases in Chile. CO/IARC Information Centre on HPV and Cancer (HPV Information Centre). 2023.

[10] WHO. Target product profiles for human papillomavirus screening tests to detect cervical pre-cancer and cancer Geneve: WHO,; 2024 https://iris.who.int/bitstream/handle/10665/379099/9789240100275-eng.pdf?sequence=1

[11] Ebisch RM, van der Horst J, Hermsen M, Rijstenberg LL, Vedder JE, Bulten J, et al. Evaluation of p16/Ki-67 dual-stained cytology as triage test for high-risk human papillomavirus-positive women. Mod Pathol. 2017;30(7):1021–31. doi: 10.1038/modpathol.2017.16 28304400

[12] Wentzensen N, Clarke MA, Bremer R, Poitras N, Tokugawa D, Goldhoff PE, et al. Clinical evaluation of human papillomavirus screening with p16/Ki-67 dual stain triage in a large organized cervical cancer screening program. JAMA Intern Med. 2019;179(7):881–8. doi: 10.1001/jamainternmed.2019.0306 31081870 PMC6515572

[13] Wright TC, Behrens CM, Ranger-Moore J, Rehm S, Sharma A, Stoler MH, et al. Triaging HPV-positive women with p16/Ki-67 dual-stained cytology: Results from a sub-study nested into the ATHENA trial. Gynecol Oncol. 2017;144(1):51–6. doi: 10.1016/j.ygyno.2016.10.031 28094038

[14] Wright TC, Stoler MH, Ranger-Moore J, Fang Q, Volkir P, Safaeian M, et al. Clinical validation of p16/Ki-67 dual-stained cytology triage of HPV-positive women: Results from the IMPACT trial. Int J Cancer. 2022;150(3):461–71. doi: 10.1002/ijc.33812 34536311 PMC9293341

[15] Clarke MA, Wentzensen N, Perkins RB, Garcia F, Arrindell D, Chelmow D, et al. Recommendations for use of p16/Ki67 dual stain for management of individuals testing positive for human papillomavirus. J Low Genit Tract Dis. 2024;28(2):124–30. doi: 10.1097/LGT.0000000000000802 38446575 PMC11331430

[16] Soto Fuentealba MF, Bustamante Saavedra C, Salazar Vergara E, Robles Pantoja J. Virus Papiloma Humano de alto riesgo y su relación con lesiones cervicales premalignas, entre los años 2020-2022, Chile. Rev Conflu. 2024;7. doi: 10.52611/confluencia.2024.1089

[17] Harper DM, Anderson RJ, Baker E, Yu TM. Cost-effectiveness of p16/Ki-67 dual-stained cytology reflex following co-testing with hrHPV genotyping for cervical cancer screening. Cancer Prev Res (Phila). 2023;16(7):393–404. 37210751 10.1158/1940-6207.CAPR-22-0455 PMC10320467

[18] MINSAL. Guías clínicas AUGE. Cáncer cérvico uterino. Ministerio de Salud de Chile: Subsecretaría de Salud Pública. División de prevención y control de enfermedades. Departamento manejo integral del cáncer y otros tumores. 2015.

[19] Safaeian M, Wright TC, Stoler MH, Ranger-Moore J, Rehm S, Aslam S, et al. The improving primary screening and colposcopy triage trial: Human papillomavirus, cervical cytology, and histopathologic results from the baseline and 1-year follow-up phase. Am J Obstet Gynecol. 2021;225(3):278.e1-278.e16. doi: 10.1016/j.ajog.2021.03.047 33852886

[20] Saville M, Sultana F, Malloy MJ, Velentzis LS, Caruana M, Ip ELO, et al. Clinical Validation of the cobas HPV Test on the cobas 6800 System for the Purpose of Cervical Screening. J Clin Microbiol. 2019;57(2):e01239-18. doi: 10.1128/JCM.01239-18 30463896 PMC6355513

[21] Mehta N, Keung MHT, Pineda E, Lynn E, Fetene D, Lee A, et al. Clinical validation of the Roche cobas HPV test on the Roche cobas 5800 system for the purpose of cervical screening. Microbiol Spectr. 2024;12(10):e0149324. doi: 10.1128/spectrum.01493-24 39258912 PMC11448194

[22] Wentzensen N, Walker JL, Gold MA, Smith KM, Zuna RE, Mathews C, et al. Multiple biopsies and detection of cervical cancer precursors at colposcopy. J Clin Oncol. 2015;33(1):83–9. doi: 10.1200/JCO.2014.55.9948 25422481 PMC4268255

[23] Del Mistro A, Matteucci M, Insacco EA, Onnis G, Da Re F, Baboci L, et al. Long-Term clinical outcome after treatment for high-grade cervical lesions: A retrospective monoinstitutional cohort study. Biomed Res Int. 2015;2015:984528. doi: 10.1155/2015/984528 26180819 PMC4477134

[24] Ferris DG, Brown DR, Giuliano AR, Myers E, Joura EA, Garland SM, et al. Prevalence, incidence, and natural history of HPV infection in adult women ages 24 to 45 participating in a vaccine trial. Papillomavirus Res. 2020;10:100202. doi: 10.1016/j.pvr.2020.100202 32464334 PMC7453107

[25] Bulkmans NWJ, Berkhof J, Bulk S, Bleeker MCG, van Kemenade FJ, Rozendaal L, et al. High-risk HPV type-specific clearance rates in cervical screening. Br J Cancer. 2007;96(9):1419–24. doi: 10.1038/sj.bjc.6603653 17342094 PMC2360183

[26] Insinga RP, Perez G, Wheeler CM, Koutsky LA, Garland SM, Leodolter S, et al. Incident cervical HPV infections in young women: Transition probabilities for CIN and infection clearance. Cancer Epidemiol Biomarkers Prev. 2011;20(2):287–96. doi: 10.1158/1055-9965.EPI-10-0791 21300618

[27] Khan MJ, Castle PE, Lorincz AT, Wacholder S, Sherman M, Scott DR, et al. The elevated 10-year risk of cervical precancer and cancer in women with human papillomavirus (HPV) type 16 or 18 and the possible utility of type-specific HPV testing in clinical practice. J Natl Cancer Inst. 2005;97(14):1072–9. doi: 10.1093/jnci/dji187 16030305

[28] Loopik DL, Bentley HA, Eijgenraam MN, IntHout J, Bekkers RLM, Bentley JR. The natural history of cervical intraepithelial neoplasia grades 1, 2, and 3: a systematic review and meta-analysis. J Low Genit Tract Dis. 2021;25(3):221–31. 34176914 10.1097/LGT.0000000000000604

[29] Kulasingam SL, Havrilesky L, Ghebre R, Myers ER. Screening for Cervical Cancer: A Decision Analysis for the US Preventive Services Task Force. Rockville (MD): Agency for Healthcare Research and Quality (US). 2011. 22553886

[30] Skorstengaard M, Lynge E, Suhr J, Napolitano G. Conservative management of women with cervical intraepithelial neoplasia grade 2 in Denmark: A cohort study. BJOG. 2020;127(6):729–36. doi: 10.1111/1471-0528.16081 31880054 PMC7383715

[31] McCredie MRE, Sharples KJ, Paul C, Baranyai J, Medley G, Jones RW, et al. Natural history of cervical neoplasia and risk of invasive cancer in women with cervical intraepithelial neoplasia 3: A retrospective cohort study. Lancet Oncol. 2008;9(5):425–34. doi: 10.1016/S1470-2045(08)70103-7 18407790

[32] Goldie SJ, Kohli M, Grima D, Weinstein MC, Wright TC, Bosch FX, et al. Projected clinical benefits and cost-effectiveness of a human papillomavirus 16/18 vaccine. J Natl Cancer Inst. 2004;96(8):604–15. doi: 10.1093/jnci/djh104 15100338

[33] Surveillance E. U.S. Mortality Data (1969-2023). Bethesda, MD: Surveillance Research Program, National Cancer Institute. 2019. https://seer.cancer.gov/statistics-network/explorer/

[34] Chuck A. Cost-effectiveness of 21 alternative cervical cancer screening strategies. Value Health. 2010;13(2):169–79. doi: 10.1111/j.1524-4733.2009.00611.x 19804436

[35] de Kok IMCM, Korfage IJ, van den Hout WB, Helmerhorst TJM, Habbema JDF, Essink-Bot M-L, et al. Quality of life assumptions determine which cervical cancer screening strategies are cost-effective. Int J Cancer. 2018;142(11):2383–93. doi: 10.1002/ijc.31265 29349795

[36] Departamento de Economía de la Salud Estudio Verificación del Costo Esperado Individual Promedio por Beneficiario del Conjunto Priorizado de Problemas de Salud con Garantías Explícitas (EVC-2021) 2022 [cited 2025 20 June]. http://desal.minsal.cl/estudio-verificacion-del-costo-esperado-individual-promedio-por-beneficiario-del-conjunto-priorizado-de-problemas-de-salud-con-garantias-explicitas-evc-2021/?preview=true

[37] Fondo Nacional de Salud (Fonasa). Modalidad de Atención Institucional: Arancel MAI Fonasa 2024. https://www.fonasa.cl/sites/fonasa/prestadores/modalidad-atencion-institucional. Accessed 2025 June 20.

[38] Central de Abastecimiento del Sistema Nacional de Servicios de Salud (Cenabast). Reporte de Compras históricas de Cenabast 2025 [cited 2025 20 April]. https://www.cenabast.cl/compras-cenabast/

[39] Mercado Público. https://www.mercadopublico.cl/Home. 2025. Accessed 2025 April 20.

[40] MINSAL. Guía metodológica para la evaluación económica de intervenciones en salud en chile. Ministerio de Salud de Chile; 2013.

[41] Ministerio de Salud de Chile. Guía clínica. Cáncer cervicouterino. Santiago: Ministerio de Salud de Chile. 2015.

[42] Ministerio deSalud de Chile. Problema de Salud AUGE N°03. Cáncer Cérvico Uterino. Recomendacioens vigentes de la guía anterior. 2020 https://diprece.minsal.cl/garantias-explicitas-en-salud-auge-o-ges/cancer-cervico-uterino/recomendaciones-vigentes-de-la-guia-anterior/

[43] Ferreccio C, Corvalán A, Margozzini P, Viviani P, González C, Aguilera X, et al. Baseline assessment of prevalence and geographical distribution of HPV types in Chile using self-collected vaginal samples. BMC Public Health. 2008;8:78. doi: 10.1186/1471-2458-8-78 18304362 PMC2291464

[44] Departamento de Estadísticas e Información de Salud. Datos abiertos. Serie REM. https://deis.minsal.cl/#datosabiertos. Accessed 2023 October 1.

[45] Campos NG, Mvundura M, Jeronimo J, Holme F, Vodicka E, Kim JJ. Cost-effectiveness of HPV-based cervical cancer screening in the public health system in Nicaragua. BMJ Open. 2017;7(6):e015048. doi: 10.1136/bmjopen-2016-015048 28619772 PMC5623348

[46] Reed N, Balega J, Barwick T, Buckley L, Burton K, Eminowicz G, et al. British Gynaecological Cancer Society (BGCS) cervical cancer guidelines: Recommendations for practice. Eur J Obstet Gynecol Reprod Biol. 2021;256:433–65. doi: 10.1016/j.ejogrb.2020.08.020 33143928

[47] Thrall MJ, McCarthy E, Mito JK, Rao J, Clinical Practice Committee of the American Society of Cytopathology. Triage options for positive high-risk HPV results from HPV-based cervical cancer screening: A review of the potential alternatives to Papanicolaou test cytology. J Am Soc Cytopathol. 2025;14(1):11–22. doi: 10.1016/j.jasc.2024.09.003 39395892

[48] Sepodes B, Rebelo T, Santos F, Oliveira D, Catalão C, Águas F, et al. Optimization of HPV-positive women triage with p16/Ki67 dual staining cytology in an organized cervical cancer screening program in the center region of Portugal. Eur J Obstet Gynecol Reprod Biol. 2024;302:111–5. doi: 10.1016/j.ejogrb.2024.09.003 39244854

[49] Ramírez AT, Valls J, Baena A, Rojas FD, Ramírez K, Álvarez R, et al. Performance of cervical cytology and HPV testing for primary cervical cancer screening in Latin America: an analysis within the ESTAMPA study. Lancet Reg Health Am. 2023;26:100593. doi: 10.1016/j.lana.2023.100593 37766799 PMC10520426

[50] Valls J, Baena A, Venegas G, Celis M, González M, Sosa C, et al. Performance of standardised colposcopy to detect cervical precancer and cancer for triage of women testing positive for human papillomavirus: Results from the ESTAMPA multicentric screening study. Lancet Glob Health. 2023;11(3):e350–60. doi: 10.1016/S2214-109X(22)00545-9 36796982 PMC10020136

[51] Correa RM, Baena A, Valls J, Colucci MC, Mendoza L, Rol M, et al. Distribution of human papillomavirus genotypes by severity of cervical lesions in HPV screened positive women from the ESTAMPA study in Latin America. PLoS One. 2022;17(7):e0272205. doi: 10.1371/journal.pone.0272205 35905130 PMC9337688

[52] Denwood M, Nielsen SS, Olsen A, Jones HE, Coffeng LE, Landfried G, et al. All that glitters is not gold: An interpretive framework for diagnostic test evaluation using Ascaris lumbricoides as a conceptual example. PLoS Negl Trop Dis. 2024;18(9):e0012481. doi: 10.1371/journal.pntd.0012481 39325683 PMC11426498

[53] Aitken CA, van Agt HME, Siebers AG, van Kemenade FJ, Niesters HGM, Melchers WJG, et al. Introduction of primary screening using high-risk HPV DNA detection in the Dutch cervical cancer screening programme: A population-based cohort study. BMC Med. 2019;17(1):228. doi: 10.1186/s12916-019-1460-0 31829241 PMC6907114

[54] Department of Health and Social Care. Impact Assessment. Decision to extend cervical screenings intervals for high-risk HPV negative individuals aged 25 to 49, creating equal screening intervals for all ages. 2024.

[55] World Health Organization (WHO). Global strategy to accelerate the elimination of cervical cancer as a public health problem 2020. https://www.who.int/publications/i/item/9789240014107

[56] World Health Organization (WHO). WHO guideline for screening and treatment of cervical pre-cancer lesions for cervical cancer prevention. 2021. Geneva. https://www.who.int/publications/i/item/9789240091658 34314129

