# Supplementary material for: Cost-effectiveness of primary HPV genotyping and dual-stain or cytology reflex testing versus cytology-based screening for cervical cancer in Chile
Source: PLoS One. 2026 Mar 4;21(3):e0332010. doi: 10.1371/journal.pone.0332010 (PMC12959674; doi:10.1371/journal.pone.0332010)
Supplement: S1 File — S1 describes the estimation of age-specific high-risk HPV prevalence based on Chilean national data and its disaggregation into HPV 16/18 and other high-risk genotypes. (DOCX) [file pone.0332010.s001.docx]

**S1. Estimation of age-specific prevalence of HPV genotypes**

The estimation of age-specific prevalence of human papillomavirus (HPV) genotypes was primarily based on the study by Ferreccio et al. (2008) (1), which served as the main data source to inform the model. This study was selected due to its methodological strengths: it was embedded within the Chilean National Health Survey (Encuesta Nacional de Salud, ENS), providing nationally representative estimates, and employed a robust and standardized protocol for HPV genotyping, thereby minimizing the likelihood of false-positive results.

The age-specific distribution of high-risk HPV (hrHPV) prevalence was extracted from graphical data reported in the original publication. Using WebPlotDigitizer, an online tool for digitizing data from images (https://automeris.io/), we digitized the prevalence curve and obtained point estimates corresponding to the midpoints of each age group. For instance, for the 25–29 age group, the estimate at age 27 was extracted. These values were then assigned as age-specific hrHPV prevalence inputs in the model.

To disaggregate total hrHPV prevalence into genotype-specific categories, we used data from Table 2 of Ferreccio et al. (2008). This table reports the distribution of hrHPV-positive cases by genotype, indicating that 23.97% corresponded to genotypes HPV 16/18, while the remaining 76.03% were attributable to other high-risk genotypes. We assumed that these proportions remained constant across all age groups, in the absence of stratified genotype data.

Under this assumption, genotype-specific prevalence for each age group was calculated using the following formulas:

$$P_{16/18} \left( a \right)= P_{hrHPV} \left( a \right) x 0.2397$$

$$P_{other 12} \left( a \right)= P_{hrHPV} \left( a \right) x 0.7603$$

Where P_hrHPV_(a) denotes the total high-risk HPV prevalence at age a. The resulting values for genotype-specific prevalence are reported in Table 1 of the main manuscript.

# **References**

1. Ferreccio C, Corvalán A, Margozzini P, Viviani P, González C, Aguilera X, et al. Baseline assessment of prevalence and geographical distribution of HPV types in Chile using self-collected vaginal samples. BMC Public Health. 2008;8(1):78.
