# Supplementary material for: Cost-effectiveness of primary HPV genotyping and dual-stain or cytology reflex testing versus cytology-based screening for cervical cancer in Chile
Source: PLoS One. 2026 Mar 4;21(3):e0332010. doi: 10.1371/journal.pone.0332010 (PMC12959674; doi:10.1371/journal.pone.0332010)
Supplement: S2 File — S2 describes the estimation of age- and genotype-specific distributions of cervical histological lesions by combining published genotype proportions with Chilean administrative data and mapping Bethesda classifications to CIN categories. (DOCX) [file pone.0332010.s002.docx]

# **S2. Estimation of Histological Lesions by Genotype and Age**

The estimation of histological lesion distribution by HPV genotype and age was primarily based on data reported by Brebi et al. (2017) (1). This study was selected due to its methodological strengths: cytological abnormalities were confirmed by colposcopy, and the genotyping protocol used closely resembled that of Ferreccio et al. (2008) (2), ensuring greater comparability and reliability of results.

From Table 1 in Brebi et al., we calculated the proportion of histological outcomes by genotype. Among women with normal findings, 2.6% and 3.9% were positive for genotypes 16/18 and other high-risk (hrHPV) types, respectively. For low-grade lesions (CIN-1), these proportions were 52.2% (HPV 16/18) and 26.9% (other hrHPV); for high-grade lesions (combined CIN-2 and CIN-3), the proportions were 32.7% and 16.0%, respectively. For invasive cervical cancer (ICC), the proportions were 79.2% for HPV 16/18 and 16.7% for other hrHPV genotypes.

Because Brebi et al. classified histological outcomes according to the Bethesda system, it was necessary to map these to the CIN classification (CIN-1, CIN-2, CIN-3) using the correspondence reported by Alrajjal et al. (2021) (3). To further distinguish between CIN-2 and CIN-3 within high-grade lesions (HSIL), we used data from the Chilean Resumen Estadístico Mensual (REM), which indicates that 56.7% of HSIL cases are CIN-2 and 43.3% are CIN-3 (4).

Using this mapping, we estimated the age-specific distribution of histological lesions by HPV genotype by combining the proportions from Brebi et al. with age-stratified lesion counts reported in the REM database. The resulting distribution is shown below:

| Age Group | CIN-1 | CIN-2 | CIN-3 | ICC | No lesion | Total |
| --- | --- | --- | --- | --- | --- | --- |
| HPV 16/18 |  |  |  |  |  |  |
| 25–29 | 1,135 | 291 | 136 | 7 | 4,663 | 6,232 |
| 30–39 | 1,683 | 548 | 341 | 119 | 8,784 | 11,475 |
| 40–49 | 1,067 | 331 | 203 | 135 | 7,836 | 9,572 |
| 50–59 | 678 | 160 | 111 | 131 | 7,512 | 8,593 |
| 60–65 | 220 | 56 | 41 | 54 | 2,993 | 3,364 |
| Age Group | **CIN-1** | **CIN-2** | **CIN-3** | **ICC** | **No lesion** | **Total** |
| Other hrHPV |  |  |  |  |  |  |
| 25–29 | 585 | 142 | 66 | 2 | 6994 | 7,789 |
| 30–39 | 867 | 267 | 166 | 25 | 13,176 | 14,502 |
| 40–49 | 550 | 161 | 99 | 29 | 11,755 | 12,593 |
| 50–59 | 350 | 78 | 54 | 28 | 11,268 | 11,777 |
| 60–65 | 113 | 27 | 20 | 11 | 4,489 | 4,661 |

Lesion counts by age group and genotype were derived by multiplying the REM-reported histological lesion totals by the genotype-specific proportions from Brebi et al. These were then normalized to obtain final estimates of histological lesion distribution by genotype and age.

Although REM data reflect administrative records for a broad and heterogeneous population, they have limitations, including a lack of clarity on whether histological confirmations stemmed from abnormal cytology or from hrHPV test results. We also considered using the IMPACT study as an alternative data source, given its large sample size (n = 34,087) and prospective design (5). However, we prioritized REM due to its greater relevance to the Chilean public healthcare context. Final proportions were normalized and incorporated into the model as age- and genotype-specific estimates of histological outcomes.

# **References**

1. Brebi P, Ili CG, Andana A, Menzel D, Lopez J, Guzman P, et al. Frequency of Human papillomavirus in women attending cervical cancer screening program in Chile. BMC Cancer. 2017;17(1):518.

2. Ferreccio C, Corvalán A, Margozzini P, Viviani P, González C, Aguilera X, et al. Baseline assessment of prevalence and geographical distribution of HPV types in Chile using self-collected vaginal samples. BMC Public Health. 2008;8(1):78.

3. Alrajjal A, Pansare V, Choudhury MSR, Khan MYA, Shidham VB. Squamous intraepithelial lesions (SIL: LSIL, HSIL, ASCUS, ASC-H, LSIL-H) of Uterine Cervix and Bethesda System. CytoJournal. 2021;18:16.

4. Departamento de Estadísticas e Información de Salud. Datos abiertos. Serie REM Ministerio de Salud de Chile: Departamento de Estadísticas e Información de Salud; 2024 [Available from: <https://deis.minsal.cl/#datosabiertos>.

5. Safaeian M, Wright TC, Stoler MH, Ranger-Moore J, Rehm S, Aslam S, et al. The IMproving Primary Screening And Colposcopy Triage trial: human papillomavirus, cervical cytology, and histopathologic results from the baseline and 1-year follow-up phase. American Journal of Obstetrics and Gynecology. 2021;225(3):278.e1-.e16.
